# Effective management of a retropharyngeal abscess using endoscopic complete-layer resection and drainage

**DOI:** 10.1055/a-2197-9172

**Published:** 2023-11-20

**Authors:** Xu Shan, Zhang Tao, Tao Kong, Jie Liu, Qingyu Zeng

**Affiliations:** 1 Department of Gastroenterology, Nanchong Central Hospital, The Second Clinical Medical College, North Sichuan Medical College, Nanchong, China; 2 Department of Gastroenterology, Nanchong Central Hospital, The Second Clinical Medical College, North Sichuan Medical College, Nanchong, China

Retropharyngeal abscess, a rare complication of foreign body ingestion that is usually associated with trauma to the retropharyngeal wall 1 2 , is also a life-threatening medical and surgical emergency, which usually requires incision and drainage 3 . Foreign bodies such as fish bones are the most common traumatic cause of retropharyngeal abscess 4 . We present a case to illustrate endoscopic complete-layer resection with drainage as a safe and effective method of treating retropharyngeal abscesses.

A 72-year-old man presented with fever and pain on swallowing for 2 days; the patient had swallowed a fish bone 20 days earlier. Computed tomography showed a retropharyngeal abscess ( Fig. 1 ). Gastroscopy revealed swollen left retropharyngeal tissue and a white pus point ( Fig. 2 a). Advancing the gastroscope through the left pharyngeal and piriform fossa was difficult ( Video 1 ). A large volume of pus was drained following the dissection of the pus point with a dual knife. Following the near-complete outflow of pus, an insulation-tipped knife was used to dissect the entire thickness of the swollen tissue, expose the abscess cavity, and ensure complete drainage. The abscess cavity contained minimal pus ( Fig. 2 b). A nasogastric tube was placed into the stomach to provide enteral nutrition.

**Fig. 1:**
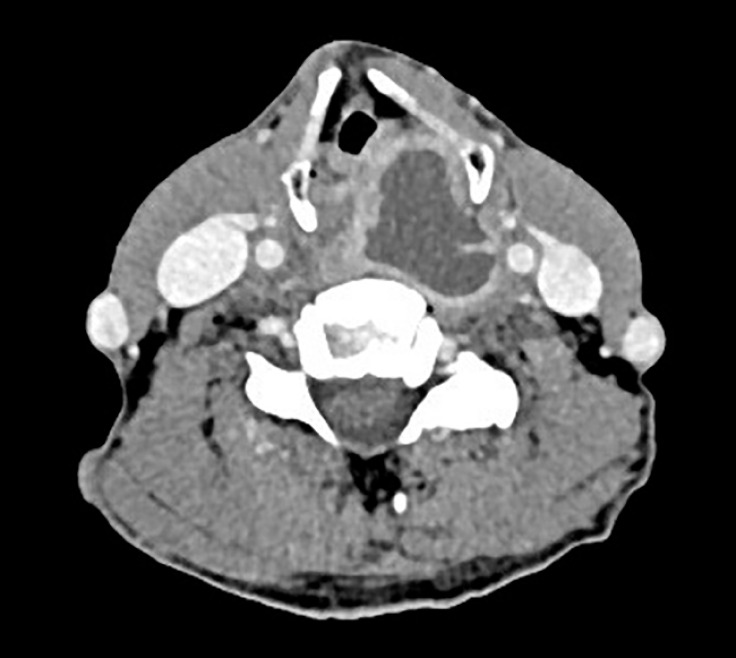
Computed tomography image, showing left retropharyngeal abscess with suspected separated diaphragm.

**Fig. 2:**
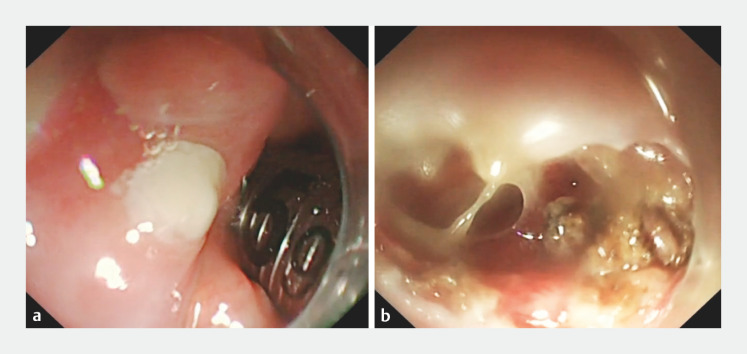
Endoscopic view. **a** Pus point on the retropharyngeal abscess. **b** Exposed abscess cavity after endoscopic complete-layer resection.

Effective management of a retropharyngeal abscess by endoscopic complete-layer resection and drainage.Video 1

Computed tomography on postintervention Day 2 revealed an empty abscess cavity ( Fig. 3 a). Computed tomography 2 weeks after intervention revealed normal left retropharyngeal tissue ( Fig. 3 b).

**Fig. 3:**
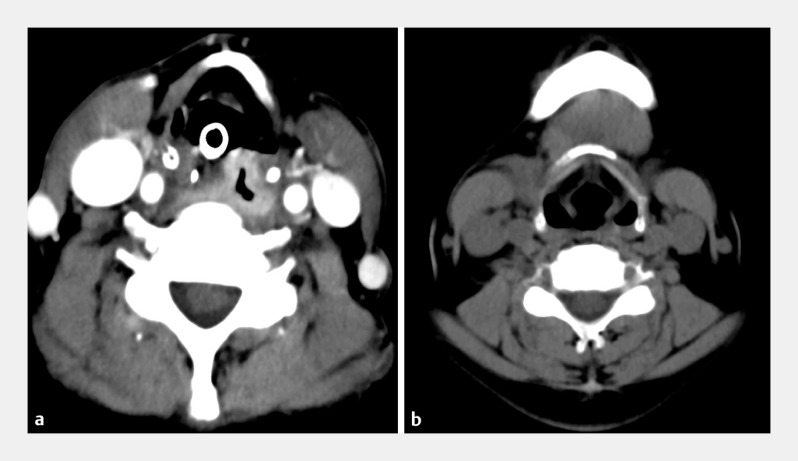
Computed tomography. **a** Image 2 days after intervention, showing completely drained left retropharyngeal abscess. **b** Image 2 weeks after intervention, showing normal retropharyngeal tissue.

Endoscopy_UCTN_Code_TTT_1AS_2AG

## Notes

Endoscopy E-Videos https://eref.thieme.de/e-videos
*E-Videos* is an open access online section of the journal *Endoscopy* , reporting on interesting cases and new techniques in gastroenterological endoscopy. All papers include a high-quality video and are published with a Creative Commons CC-BY license. Endoscopy E-Videos qualify for HINARI discounts and waivers and eligibility is automatically checked during the submission process. We grant 100% waivers to articles whose corresponding authors are based in Group A countries and 50% waivers to those who are based in Group B countries as classified by Research4Life (see: https://www.research4life.org/access/eligibility/ ). This section has its own submission website at https://mc.manuscriptcentral.com/e-videos .
